# 

**Published:** 1891-11-14

**Authors:** 


**Notices of Births, Deaths, and Marriages**, are inserted in this column at a charge of 2s. 6d. for an announcement not exceeding four lines.

### NOTICE TO CORRESPONDENTS.

**All MS., Letters, Books for Review, and other matters intended for the Editor should be addressed The Editor, The Lodge, Porchester Square, London, W.**

**The Editor cannot undertake to return rejected M.S., even when accompanied by stamped directed envelope.**

**Notice to Advertisers and Subscribers.**

**All Advertisements, Orders for Copies of The Hospital, and Business Communications should be addressed to The Publisher,** *not to the Editor,* **The Hospital, 140, Strand, W.C.**

**The Cost of Subscription, post free, is as follows:—Per week, 1½d.; per quarter, 1s. 8d.; per half-year, 3s. 3d.; per annum, 6s. 6d. Foreign:—Per annum, 8s. 8d.; payable, in all cases, in advance.**

**"Burdett's Hospital Annual," 1891—1892.**

Third year of publication. 600 pp. Boards, gilt lettering. A most complete guide to British and Colonial Hospitals, Dispensaries, Nursing and Convalescent Institutions, and Asylums. Price 3s. 6d. Published by The Hospital (Limited), 140, Strand, W.C.

**NOTICE.—The Index for Vol. X. (ending September, 1891), is on sale at the Office of this Journal, price 2½d., post free.**

### General Charities.

[This Column is devoted to an account of work of charitable institutions other than Medical Charities. The Editor will be glad to receive reports or news of work at 140, Strand. *Philanthropy* should be plainly written on the envelope.]

Manchester, following the example generously set by the *Yorkshire Post*, has made a collection on behalf of the Lifeboat Society, so it is to be hoped that the deficit to which we recently alluded will soon be more than made up.

A new Orphan Home has been opened in connection with the **GLASGOW INSTITUTION FOR ORPHAN AND DESTITUTE GIRLS**, in which accommodation is provided for 60 girls. The Institution was founded some sixty years ago for the purpose of giving an elementary education to destitute and orphan girls, and of training them for domestic service.

A novel method for raising money for charities has recently been adopted in Melbourne, where an exhibition of pin-cushions was held, the proceeds of which were devoted to the District Nursing Association. Prizes were given for the most artistically-arranged pin-cushions. An additional attraction was a cake fair, and the novelty attracted a large number of people. Whether the majority went to see the pin-cushions or to eat the cake is not officially stated.

A "Good Samaritan" office is to be opened near the Monument, in the Rectory of St. Mary-at-Hill, City, by the **CHURCH ARMY**. The object of the institution is to assist into employment men who are above the class which frequents casual wards. This new departure, which we hope will meet with all success, is to be carried on like the rest of the Social Scheme of the Church Army. There will be classes for instruction in type-writing, and a fee of one penny, which in some cases can be remitted, will be charged for each lesson.

The financial statement which has just been issued by the secretaries of the **SOCIETY FOR PROMOTING CHRISTIAN KNOWLEDGE** is far from satisfactory. The charitable operations of the Society cover a large area and require a large sum; in fact, the normal income is £40,000, the greater part of which is derived from investments, one-third about being raised by subscription. The subscriptions have latterly shown a considerable falling off, and if this steady decrease continues, the Society will undoubtedly have to curtail its work.

Appeals are now being issued from various quarters for subscriptions in aid of funds for providing dinners for the poor children during the winter months. Those who have seen the misery of some of these poor little neglected and hungry bodies can only fully appreciate how much good can be done in this direction. Unfortunately, though supported by the best intentions, the desired good is not always easy to accomplish. Unless the distribution of the meals is well managed, the wrong children get the food, and the hungry bodies are still left to starve. When once the funds have been collected, and the food has been prepared, the inexperienced are apt to think that all that is necessary has been done, and with a light heart and indiscriminating hand they distribute to a clamouring crowd. It is essential to remember that the very ones for whom the food should be provided are too weak to press to the front and urge their claims of emptiness and suffering, and want searching out; whereas those who so often come forward with such alacrity, show by their very power to do so that they are not in such sore need as they would represent. Immense good can be done in the direction of providing food for poor children, but, undoubtedly, equal harm can be done by want of due discrimination.

In *Night and Day* Dr. Barnardo makes the following statement: "I have been much tried during the past two years or so by the conduct of some delinquents whom it is hard to bring to book, and who still persevere, with an audacity worthy of a better cause, in their nefarious pursuits. These people go from house to house to collect money, professedly on my behalf, and do the children's cause much damage. Of course they are not furnished with any proper authorisation, and in a vast number of cases succeed in deceiving kindly-disposed and charitable people, for it need hardly be said the money so collected never reaches my hands. One enterprising lady actually has had a card printed. She very cleverly defends herself from suspicion in the following way: On one occasion she sent me, as if she were a benevolent stranger, the sum of 6s., which she said she had collected from her friends. Of course, I sent her an official receipt, setting forth her name, and stating that it was the result of a collection. This official receipt is treasured accordingly, and is shown by her to anyone who may have suspicion of her proceedings. . . . As a matter of fact, this person must have gained very many pounds, and as it is impossible to deal with her unless we can get someone to prosecute, the mischief goes on indefinitely." This is certainly a novel way of making money, and sounds lucrative. There are many people who have a strong objection to the authorised system of collecting by cards; that it is open to abuse there has never been a doubt, except in the minds of blind enthusiasts, and Dr. Barnardo gives an unpleasant instance of such abuse. It would be as well if the names of the donors were entered, with their addresses, on all collecting cards.

### Scraps and Gleanings.

A Provident Dispensary is projected for Halifax.

The Hospital Saturday collection at Coventry has amounted to £1,313 this year.

The expenditure at the Taunton and Somerset Hospital shows an excess of £570 over income.

The library of the Paris Faculty of Medicine has been removed to new and extensive quarters.

In South Lincolnshire the influenza epidemic has made its reappearance, and a large number of people are affected.

Cholera is spreading rapidly at Damascus, where in one week 182 cases, of which 90 ended fatally, have been recorded.

The first Hospital Saturday collection in Birmingham only produced £104. This year it is able to show receipts to the amount of £4,234.

Kettering is awaking from its apathy in respect to the proposed hospital, and steps are now being taken for the serious consideration of the scheme.

It is proposed that the Hospital Saturday collection about to be established at Cork, should be worked on the same system as obtains in Birmingham.

Miss Urania Latham, daughter of the late Rev. E. Latham, of Repton and Matlock, has gained the Entrance Scholarship of £30 at the London School of Medicine.

The High Wycombe Cottage Hospital bears witness to able management and popularity. It is in a flourishing financial condition, and additions are shortly to be made to the Institution.

Mr. Ballantine, M.P., speaking at Coventry last week, announced his intention of introducing next session a Bill to suspend vaccination prosecutions pending the issue of the report of the Royal Commission.

The Cheltenham Hospital Collection in the churches cannot be looked upon as altogether satisfactory, inasmuch as receipts do not come up to the standard of former years. The collection nevertheless amounts to £484.

A French paper recommends potato as a conserving medium for steel pens. Its components are stated to neutralise the acidity of the ink. This opens a cheap and novel field for useful presents for the writing table!

A pleasant gathering assembled on the 1st of this month, at the Women and Children's Hospital, Cork, when Lady Colthurst laid the foundation-stone of a new bath-room wing to be added to the institution.

A tragedy lately occurred at the Lunatic Asylum, Rennes, France. One of the inmates was killed by one of the attendants in a fit of rage. The post-mortem revealed traces of more than forty extensive bruises caused by the foot and stick.

A violent outbreak of influenza has appeared in Angoulême and other places in Charente. No fewer than 30 persons have been prostrated at Larochefoucauld; and at the Seminary at Richemont six masters and over sixty pupils are on the sick list.

A man suffering from leprosy was taken up for breaking a shop window at Edmonton. He was on his way to the infirmary of the Strand Union Workhouse, and the magistrate before whom he appeared discharged him on condition that he immediately repaired to his destination.

At the Quarterly Court of the Radcliffe Infirmary, Oxford, an alteration was proposed to be made in the wording of the rules respecting the admission of patients. This alteration, if carried, will remove the words "objects of charity," now objected to, from the admission formulæ.

Miss Baxter, Lady Superintendent of the Cork Women and Children's Hospital, received much praise at the recent gathering at the Hospital. To her exertions the contemplated additions to the institution are due. Her nurses are admirably trained, and give satisfaction wherever they go.

Miss Emma Villers-Wilkes, late of Edgbaston, has by her will bequeathed (free of legacy duty) to the medical charities of Birmingham the following legacies: The General Hospital, £1,000; the General Dispensary, £1,000; the Children's Hospital, £500; the Birmingham and Midland Ear and Throat Hospital, £500.

The good people of Hebburn may be said to have placed the "cart before the horse." This is the second "hospital demonstration and collection" that has been held "in the place where the hospital is not." Surely with such enthusiasm the only thing needful, *i.e.*, a hospital, will soon be forthcoming.

Mr. William Rowland, of Gateshead-on-Tyne, having read of the Salford Royal Hospital, and the statements made regarding its financial position, waited on the Treasurer at the hospital and presented him with a £100 note. Mr. Rowland formerly lived in Salford, and this is the second donation of £100 that he has made.

A telegram from Lebanon, Indiana, states that a young girl named Neese was persuaded, a fortnight ago, to smoke a cigarette, and immediately afterwards became very ill, and has since died from nicotine poisoning, it is stated. Here is a fact for those who look askance at the increasing habit of smoking amongst ladies.

A misadventure occurred at the London Hospital last week, the victim of which has since died. A woman visiting a patient administered a draught of diluted carbolic acid to a patient suffering from dropsy, under the impression that it was water. It was not proved that the poison was the immediate cause of death.

The annual report of the Salop Infirmary is not encouraging. The income of the institution, which has been falling off for some years, is lower this year than ever. The deficiency is principally in annual subscriptions, and everyone knows that a great deal of energy on the part of the direction is necessary to maintain a steady return from this quarter.

It seems a difficult matter to quell the fears of people dwelling in the vicinity of the proposed site of the Hospital for Consumptives for Belfast, about to be erected. One correspondent of the local papers, whether from interested motives or not we do not know, suggests that the proximity of the "Ulster Cricket Club" to the suggested site will be bad for the Hospital.

### THE STUDENT OF MEDICINE.

[All communications for this Supplement should be addressed to The Editor of The Hospital, at 140, Strand, with the words, "Students' Column," written in the left hand top corner of the envelope.]

### STUDENTS' MEDICAL SOCIETIES.

**Owens College, Manchester.—Medical Students' Debating Society** (October 19).

"Mr. William Thorburn, M.D., F.R.C.S., started a discussion on "Methods of Obtaining Medical Evidence in Legal Investigations." Mr. Thorburn introduced his subject by calling attention to the present highly unsatisfactory position of medical witnesses in civil and criminal investigations. The frequent conflict of medical evidence, often on the most material points, has constantly been made a cause of reproach to the profession, both by lawyers and by the laity. That there are some grounds for this reproach cannot be denied, but as it will hardly be suggested that the moral standard of medical and other scientific men, who appear as experts in courts of justice, is an exceptionally low one, we are driven to look for a serious flaw in the system of obtaining expert evidence, rather than in the characters of those who appear as witnesses. The present system of the English law-courts has two chief drawbacks, viz.: 1, that every expert is almost necessarily obliged to make a more or less *ex parte* statement, and 2, that it is often impossible for a jury, composed of laymen, to arrive at a correct mean between the two extreme views thus set before them. Instances were adduced of miscarriages of justice thus brought about. To remedy this state of things it was urged that some system should be adopted by which an opinion might be obtained independently of either of the parties concerned in a civil or criminal trial. The French and German methods of procedure were described, and, basing his remarks partly upon these, Mr. Thorburn suggested the following as the lines for future legislation: "That all medico-legal questions should be referred to a committee consisting of three experts. That these experts should be appointed either (1) as permanent officers attached to certain districts, or (2) in the event of no permanent officer being available, by the judge or other responsible official of the court concerned. This committee should be empowered to take any medical evidence tendered by either party to the action—such evidence being heard without the interposition of counsel—to examine personally the individual concerned, or any relevant material, such as food or vomited matters suspected to contain poison, and to obtain the opinion of any other specialist whom they might wish to consult. The opinion of this committee would be delivered to the court in writing, and except as regards purely legal points, would be final, and would as such be presented to the jury." In the discussion which followed the present unsatisfactory condition of affairs was generally admitted. The President, Dr. Kelynack, took exception to the plan, proposed by Mr. Thorburn, of appointing permanent officials, both as an infringement of liberty and on account of the abuses to which such a system appeared liable. Dr. Wild made a valuable contribution to the debate, and prophesied an early amendment of the existing methods. Messrs. P. McDougall, Higginson, Marshall, and Cooper also spoke. After a very hearty vote of thanks had been accorded to Mr. Thorburn for his able and interesting address, he replied, and the proceedings terminated.

### APPOINTMENTS.

At the Charing Cross Hospital Medical School, John Harold, M.R.C.S., L.R.C.P., has been appointed Demonstrator of Physiology, and D. J. Jones, M.R.C.S., L.R.C.P., surgical registrar. At the Eastern Counties Asylum for Idiots, Frederick William Hall, M.B., B.S., L.R.C.P., M.R.C.S., has been appointed resident medical attendant. At the Children's Hospital, Glebe Point, New South Wales, L. R. Huxtable, M.B., C.M. Edin., has been appointed honorary physician. At the Islington Dispensary, D. J. Jones, M.R.C.S., L.R.C.P., has been appointed resident medical officer.

### VACANCIES.

The following vacancies are announced this week, the amount of salary in each case being given after the name of the institution:

**Resident Medical Officers.**

Asylum for Idiots, Earlswood, Redhill, Surrey, Assistant Medical Officer—Salary £153 and board, &c.
Cheltenham General Hospital, Junior House Surgeon—Salary £40, with board, &c.
Clayton Hospital and Wakefield General Dispensary, Wakefield, Junior House Surgeon—Honorarium £25, with board, &c.
Dudley Dispensary. Medical Officer—Salary £130, with house.
Garlands Asylum, Carlisle, Junior Medical Assistant—Salary £80, with board.
Radcliffe Infirmary, Oxford, House Physician—Salary £80, with board, &c.

**Non-Resident Medical Officers.**

Dental Hospital of London, Leicester Square, Dental Surgeon and Assistant Dental Surgeon.

